# An overview of recent research in marine biological invasions

**DOI:** 10.1007/s00227-017-3155-4

**Published:** 2017-05-03

**Authors:** Farrah T. Chan, Elizabeta Briski

**Affiliations:** 1 Great Lakes Laboratory for Fisheries and Aquatic Sciences, Fisheries and Oceans Canada, Burlington, ON L7S 1A1 Canada; 2 GEOMAR, Helmholtz Centre for Ocean Research Kiel, Düsternbrooker Weg 20, 24105 Kiel, Germany

**Keywords:** Alien species, Aquatic ecosystems, Biodiversity, Exotic species, Nonindigenous species, Non-native species, Invasive species

## Abstract

The Topical Collection on Invasive Species includes 50 articles addressing many tenets of marine invasion ecology. The collection covers important topics relating to propagule pressure associated with transport vectors, species characteristics, attributes of recipient ecosystems, invasion genetics, biotic interactions, testing of invasion hypotheses, invasion dynamics and spread, and impacts of nonindigenous species. This article summarizes some of the collection’s highlights.

## Introduction

The introduction and establishment of nonindigenous species (NIS) beyond their natural distributional range is a key component of global environmental change (Simberloff et al. 2013). While only a small proportion of introduced NIS become invasive in the recipient habitats (Blackburn et al. 2011), their impacts can be detrimental. Invasive NIS can lead to declines or even extinctions of native species, disrupt ecosystem functions, enhance transmission of viruses and pathogens, and cause substantial damage to natural resources and ecosystem services (Simberloff et al. 2013). Consequently, considerable research has been conducted to examine the biology, ecology, and evolution of NIS (e.g., Sakai et al. 2001; Roman and Darling 2007; Lejeusne et al. 2014), characterize key transport vectors and pathways (i.e., transport means and geographic routes, respectively) (e.g., Hulme 2009; Wilson et al. 2009), identify determinants of invasion success (e.g., Kolar and Lodge 2001; Williamson 2006; Blackburn et al. 2015), forecast spatial distribution and spread (e.g., Muirhead and MacIsaac 2005; Floerl et al. 2009; Larson et al. 2014), as well as assess and predict impacts of NIS on recipient communities (e.g., Dick et al. 2013; Alexander et al. 2014; Jeschke et al. 2014). Understanding the mechanisms and patterns of biological invasions allows us to develop strategies to prevent and manage the negative effects of NIS (Pyšek and Richardson 2010), while gaining valuable insights into ecological, evolutionary, and biogeographic theories and concepts (see Lodge 1993; Sax et al. 2007; Jeschke 2014).

Research efforts in invasion ecology; however, vary across systems, with the majority of studies conducted in terrestrial habitats rather than aquatic ones (Jeschke et al. 2012; Lowry et al. 2012). Marine and coastal ecosystems worldwide are being invaded at extraordinary rates as a result of human activities such as shipping, aquaculture, fisheries, ornamental and live seafood trades, the opening and construction of canals, habitat modification, and climate change, which provide increasing opportunities for marine NIS to be introduced and subsequently established in new environments (Occhipinti-Ambrogi and Savini 2003; Molnar et al. 2008; Williams et al. 2013). Therefore, studies focusing on biological invasions in marine and coastal environments are warranted.

This Topical Collection on Invasive Species compiles 50 articles, including 44 Original Papers, four Reviews, and two Short Notes, addressing a wide array of topics in marine invasion ecology. Additionally, the collection is complemented by two Editorial Comments, a preface (Briski and Chan 2015) and this summary. The topics of these articles can be broadly categorized into eight themes: propagule pressure associated with transport vectors, species characteristics, attributes of recipient ecosystems, invasion genetics, biotic interactions, invasion hypothesis testing, invasion dynamics and spread, and impacts of nonindigenous species. This collection covers a number of nonindigenous algae, macrophytes, invertebrates, and fishes in marine and coastal ecosystems spanning from as far south as Argentina and New Zealand to as far north as the Canadian Arctic. The range of approaches used in the studies includes field surveys, field experiments, laboratory experiments, bioassays, literature reviews, meta-analyses, and mathematical modelling. Here, we briefly summarize some of the highlights of this collection, while a detailed description of each study can be found in the original paper. Although many studies addressed more than one topic, we focus on the most interesting and important findings of each publication for the sake of brevity.

## Propagule pressure associated with transport vectors

A number of theoretical and empirical studies have demonstrated that propagule pressure—the number of individuals involved in an introduction event (propagule size) and the number of introduction events (propagule number)—is the most consistent predictor of invasion success (e.g., Von Holle and Simberloff 2005; Lockwood et al. 2005; Simberloff 2009). Increased propagule size and propagule number enhance the probability of establishment of a population by decreasing environmental and demographic stochasticity, respectively (Lockwood et al. 2005; Simberloff 2009; Blackburn et al. 2015). Transport vectors influence the movement, quantity, and quality of propagules being delivered to new habitats, thereby playing a crucial role in determining the outcome of biological invasions (Lockwood et al. 2005; Hulme 2009; Wilson et al. 2009).

This collection covers two leading transport vectors of NIS in marine and coastal ecosystems: shipping (ballast water and biofouling) and aquaculture. Casas-Monroy et al. (2016) presented the first estimation of propagule pressure for viable (i.e., alive and able to reproduce) nonindigenous dinoflagellates in ballast water of commercial ships arriving at Pacific and Atlantic ports in Canada. The authors found that propagule pressure varied by transport pathway, and that ballast water exchange was not sufficient to reduce ballast-mediated invasion risk in marine and coastal habitats. Three studies evaluating the importance of ship biofouling as a vector for the introduction and spread of marine NIS reported mixed results (Chan et al. 2016; Kauano et al. 2017; van der Gaag et al. 2016). The first study examining temporal changes in biofouling assemblages on military vessels during transits in the marine Arctic recorded six potential NIS capable of surviving round trip voyages from temperate to arctic ports in Canada (Chan et al. 2016). Similarly, an experimental study found that the sailing speed and desiccation time typical of small fishing and recreation boats in southern Brazil had little effect on the survivorship of biofouling organisms (Kauano et al. 2017). In contrast, results from mesocosm experiments indicate that the nonindigenous mussels *Dreissena polymorpha* and *Mytilopsis leucophaeata* and the native mussel *Mytilus edulis* would not be capable of tolerating unfavourable salinity levels for durations typical of actual ship voyages in the North Sea (van der Gaag et al. 2016). In terms of the aquaculture vector, a laboratory study demonstrated that transplanted mussels could serve as a transport vector for *Nitzschia bizertensis*, a new toxin-producing diatom recently found in Bizerte Lagoon, Tunisia; the diatom was able to survive, regrow, and retain its toxicity following filtration and ejection as biodeposits by mussels (Bouchouicha-Smida et al. 2015).

## Species characteristics

Species characteristics such as fast growth, polyphagy, high dispersal ability, broad physiological tolerance, high genetic variability, high phenotypic plasticity, and association with humans have been proposed as common attributes of successful NIS (e.g., Sakai et al. 2001; Kolar and Lodge 2002; Jeschke and Strayer 2006; Blackburn and Jeschke 2009; Lenz et al. 2011). Articles in this collection focused on the importance of physiological tolerance to the invasion success of marine NIS. Species with the capacity to tolerate broader abiotic conditions may have a greater likelihood of surviving transport, establishing in new habitats, and expanding their introduced range (Lenz et al. 2011; Bates et al. 2013). An experimental study investigating the effects of temperature and salinity on the performance of the nonindigenous kelp *Undaria pinnatifida* and two native kelps, *Lessonia variegata* and *Ecklonia radiata*, from Tauranga Harbour, New Zealand found that the NIS generally exhibited broader tolerance to the treatments than the native ones (Bollen et al. 2016). Indeed, a separate study reported that the nonindigenous population of *U. pinnatifida* in Hauraki Gulf, New Zealand was able to tolerate temperatures much warmer than those in its native range by adjusting its growth cycle, allowing individuals to persist under conditions previously thought to be unfavourable (James and Shears 2016a).

Differential physiological tolerance between native and nonindigenous populations of a species suggests that the trait may be selected during the invasion process (Hammann et al. 2016; Schmidt et al. 2016). Comparisons of the physiological responses of native and nonindigenous populations of the seaweed *Gracilaria vermiculophylla* to heat shock in common-garden experiments conducted in both the native (Qingdao, China) and introduced (Kiel, Germany) ranges suggest that the nonindigenous populations are more tolerant to heat stress than the native ones (Hammann et al. 2016). Similarly, two populations of the foraminifera *Amphistegina lobifera*—one from the Gulf of Aqaba that invaded the Red Sea during the post-glacial recolonization, and a recently-invaded Lessepsian population from the Eastern Mediterranean—exhibited exceptional thermal resistance that has no apparent adaptive relevance to the local environments (Schmidt et al. 2016).

Within a population, differences in physiological tolerance between sex and growth phases may have implications for invasion success. Pennoyer et al. (2016) reported that individuals in the green colour phase of the nonindigenous European green crab *Carcinus maenas* from Maine, USA typically performed better than those in the red colour phase under conditions of low salinity, and that females often outperformed males in their respective colour phases.

## Attributes of recipient ecosystems

Once arrived at new habitats, successful NIS must survive ambient environmental conditions and establish a self-sustaining population (Blackburn et al. 2011). Ecosystems that are susceptible to invasions tend to have environmental conditions similar to those of the native habitat of invading NIS, high environmental heterogeneity, a history of habitat disturbance, low species diversity, and few natural enemies (e.g., Levine et al. 2004; Fridley et al. 2007; Melbourne et al. 2007; Herborg et al. 2007; Clark and Johnston 2011). In fact, a review study identified a network of anthropogenic (e.g., physical disturbance, increased sedimentation, eutrophication, and fishing), abiotic (e.g., substrate complexity and water movement), and biotic (e.g., the presence of canopy macrophytes, alga turfs, other NIS, and herbivory) variables that regulate the spread of the nonindigenous green macroalgae *Caulerpa cylindracea* in the Mediterranean Sea (Piazzi et al. 2016).

Human-modified habitats such as shipping ports, marinas, and aquaculture sites may serve as hotspots for marine invasion owing to high propagule supply and/or abiotic features that promote NIS establishment. An examination of invasion patterns in shipping ports on the Atlantic and Pacific coasts of Canada revealed that latitude, salinity, sediment type, and human populations were strongly related to NIS establishment (Choi et al. 2016). While summer water temperature and cargo shipping traffic explained the majority of variability in the number of fouling NIS established in coastal regions of the United States (Lord et al. 2015), a study investigating the association between the presence of NIS and physical features of marinas in the United Kingdom identified freshwater input, marina entrance width, and seawall length as significant predictors of NIS occurrence (Foster et al. 2016). Populations of the nonindigenous kelp *U. pinnatifida* on mussel farms were more prolific, with longer annual presences and greater reproductive capacity than those on natural reefs, because aquaculture sites provided the optimal environmental conditions (e.g., high water clarity and great water motion) for NIS establishment (James and Shears 2016b).

While classical invasion theory suggests that disturbance promotes invasion by freeing resources and reducing competition (Elton 1958; Davis et al. 2000), its influence on invasion success appears to be complex, depending on species and ecosystem properties, type and timing of disturbance, and spatial scale (e.g., Lonsdale 1999; Melbourne et al. 2007; Clark and Johnston 2011). An experimental study reported that colonization by the nonindigenous isopod *Cirolana harfordi* from Sydney Harbour, Australia was facilitated by the presence of an assemblage and influenced by the type of resident assemblage, with greater success on disturbed assemblages than undisturbed ones (Bugnot et al. 2016). The type of disturbance was important, as an alternative source of organic matter simulating the effects of disturbance occurring upstream of the study site had no influence on colonization (Bugnot et al. 2016). In contrast, results of two empirical studies conducted in New Zealand suggest that disturbance had limited effects on the recruitment of *U. pinnatifida* (Morelissen et al. 2016; South and Thomsen 2016). The timing and size of native algal cover removal did not affect the recruitment of *U. pinnatifida* on experimental plots located on a rock low-intertidal shore in central New Zealand (Morelissen et al. 2016). Similarly, while native canopy removal facilitated the recruitment of *U. pinnatifida* on experimental plots in Lyttelton Harbour, New Zealand, the nonindigenous kelp had weak and transient impacts on the native assemblages during the early stage of the invasion at a small spatial scale (South and Thomsen 2016). The study concluded that the kelp was a “passenger”, not “driver”, of ecological change (South and Thomsen 2016).

## Invasion genetics

Evolutionary processes (e.g., genetic bottleneck, genetic drift, selection, admixture, and adaptation) can strongly influence whether invading NIS can persist and proliferate in introduced environments (Sakai et al. 2001; Lee 2002; Roman and Darling 2007). Johnson et al. (2016) presented the first study that examined the genetic composition of the lionfish *Pterois volitans/miles* in the Gulf of Mexico, which suggests that the nonindigenous populations likely originated from the Caribbean and expanded rapidly after initial colonization, despite undergoing a genetic bottleneck. The first genetic study conducted for nonindigenous populations of the sponge *Paraleucilla magna* in the Mediterranean and Northeastern Atlantic revealed that the invasion success of the species could be attributed to high genetic diversity, likely owing to multiple introductions and phenotypic plasticity (Guardiola et al. 2016). A study examining the temporal genetic structure and diversity of a nonindigenous population of the ascidian *Styela plicata* in Wilmington, North Carolina, USA found that the population was maintained by a recurrent arrival of propagules from neighbouring populations, supplementing the genetic pool with new alleles after exposure to periodic floods and fluctuations in temperature and salinity (Pineda et al. 2016). Using cytochrome c oxidase subunit I barcoding sequences, Sun et al. (2017) examined the genetic divergence among global populations of the calcareous tube worm *Hydroides dianthus*. The authors demonstrated that *H. dianthus* is a species complex consisting of two phylogenetic lineages (Clades A and B). Interestingly, results of the study suggest that the native range of *H. dianthus* might be the Mediterranean, rather than the east coast of USA as previously assumed (Sun et al. 2017).

Recent advances in genomics tools can further improve our understanding of the role of evolutionary processes in determining the success of marine NIS. In a review paper, Sherman et al. (2016) discussed how genomic (DNA), transcriptomic (RNA), and epigenetic tools can be used to identify adaptive variation within and among (native and nonindigenous) populations, to uncover candidate genes responsible for certain adaptive traits, and to understand the mechanism of epigenetic variation in plastic responses to novel environments. In particular, high-throughput sequencing (HTS)-based methods have been used as tools for early detection and monitoring of marine NIS owing to their high capacity to detect species at low abundance with low associated costs (Zhan et al. 2013; Carugati et al. 2015; Simmons et al. 2016). Xiong et al. (2016) outlined the major technical issues that can lead to false negatives and false positives when employing these methods, discussed the causes and consequences of these errors, and offered solutions for future studies.

## Biotic interactions

Biotic interactions may promote or impede the establishment and spread of NIS in recipient environments via mechanisms such as competition, exploitation, facilitation, and mutualism (e.g., Simberloff and Von Holle 1999; Freestone et al. 2013; Alofs and Jackson 2014). This collection explores the effects of facilitation, kleptoparasitism, scavenging, herbivory, predation, and competition on invasion success of marine NIS. An example in which biotic interactions facilitated the establishment of a marine NIS is provided by Drouin et al. (2016). The abundance of the nonindigenous green alga *Codium fragile* ssp. *fragile* in an eelgrass habitat in Grande-Entrée Lagoon (Magdalen Islands, Eastern Canada) was positively related to the density of the native canopy-forming species *Zostera marina* (Drouin et al. 2016). The native species may be essential to the establishment of *C. fragile* by trapping sediments and algal fragments, providing substrata for anchorage (Drouin et al. 2016). In contrast, Silva et al. (2017) found that native sponges (e.g., *Iotrochota arenosa* and *Scopalina ruetzleri*) may occasionally outcompete nonindigenous *Tubastraea* corals in Ilha Grande Bay, Brazil by overgrowing them, though the most common competitive interaction among the species is contact without dominance.

The influence of biotic interactions on the invasion success of marine NIS; however, may be less straightforward. For instance, mathematical models examining the trophic interactions among the nonindigenous green crab *C. maenas* and the native dogwhelk *Nucella lapillus* foraging on *Mytilus* spp. mussels in Atlantic Canada revealed that crab kleptoparasitism (i.e., crabs taking mussels from whelks) had negative effects on whelks, whereas no significant impact on whelks was detected for crab scavenging (i.e., crabs feeding on mussels abandoned by whelks) (Quinn and Boudreau 2016). A study examining the impact of a native herbivorous reef fish, the bluespine unicornfish (*Naso unicornis*), on the growth and distribution of the nonindigenous *Gracilaria salicornia* in the Hawaii Marine Laboratory Refuge reported that the unicornfish might serve as both a control agent and a natural transport vector for *G. salicornia* (Bierwagen et al. 2017). Finally, results of laboratory experiments suggest that a complex habitat mediated the negative effects of predation on the native mud crab *Dyspanopeus sayi* by the nonindigenous European green crab *C. maenas* in Atlantic Canada (Gehrels et al. 2016).

## Invasion hypothesis testing

Several hypotheses have been proposed to explain successful invasions (Catford et al. 2009; Jeschke et al. 2012; Lau and Schultheis 2015). However, these hypotheses have been tested mostly in terrestrial systems rather than aquatic ones, and the results are inconsistent (Ricciardi and MacIsaac 2011; Jeschke et al. 2012). Studies in this collection empirically tested the enemy-release hypothesis (ERH), the novel weapon hypothesis (NWH), and the evolution of increased competitive ability (EICA) hypothesis in marine and coastal habitats. The ERH proposes that NIS are liberated from the negative effects of co-evolved natural enemies, and are thus able to establish and/or cause detrimental impacts in introduced environments (Elton 1958; Colautti et al. 2004; Prior et al. 2015). In the same vein, the NWH suggests that the success of NIS is related to their competitive, defensive, or predatory traits, which native species have never encountered before in invaded habitats (Callaway and Ridenour 2004). The EICA hypothesis states that the escape from natural enemies allows NIS to reallocate resources from defense to growth and competitive ability via evolutionary mechanisms (Blossey and Nötzold 1995). Three studies examining the feeding preference of native invertebrate grazers for nonindigenous algae (*Sargassum muticum*, *Heterosiphonia japonica*, and *U. pinnatifida*) and a number of native competitors in pair-wise and multiple-choice feeding assays found that the grazers generally preferred native algae over the nonindigenous ones because they were deterred by the chemical properties (e.g., secondary metabolites) of the invaders (Jiménez et al. 2015; Sagerman et al. 2015; Schwartz et al. 2016). These results are consistent with the ERH and EICA hypotheses. In addition, the grazers’ preference for *S. muticum* from the invaded habitat (North Sea) over the ones from the native range (Japan) could indicate a resource allocation from chemical defense to reproduction and growth, which is in line with the EICA hypothesis (Schwartz et al. 2016).

Results of two studies; however, did not support the ERH (Merella et al. 2016; Pedersen et al. 2016). Pedersen et al. (2016) demonstrated that the nonindigenous brown alga *S. muticum* was typically consumed at the same rate or faster than a range of native algae, depending on the growth rate and morphology of algal species being compared in feeding experiments. A parasitological study of the nonindigenous bluespotted cornetfish (*Fistularia commersonii*) in the Mediterranean Sea found no evident decrease of parasite richness and levels of infection in the fish (Merella et al. 2016). In fact, the species acquired new parasites while retaining a subset of natural ones in the invaded range (Merella et al. 2016).

## Invasion dynamics and spread of NIS

Analyzing invasion patterns of NIS can provide insights into the drivers and mechanisms of biological invasions (e.g., Marini et al. 2013; Ruiz et al. 2013; Gallardo and Aldridge 2015) and permit the projections of future spread (e.g., Peterson 2003; Chu et al. 2005; Herborg et al. 2007). Knowledge of current and future distributions of NIS allows resource managers to prioritise control and prevention efforts at high-risk sites. For example, an examination of temporal and spatial patterns of ascidian invasions in the continental United States and Alaska identified an invasion hotspot on the Pacific coast and ship biofouling as the primary transport vector (Simkanin et al. 2016). In addition, a review of ascidian invasions worldwide provided by Zhan et al. (2015) outlined the invasion history and impacts of nonindigenous ascidians, factors underlying the success of these invasions, and relevant regulations and management strategies that are available to prevent and control further spread. Similarly, Marchini and Cardeccia (2017) presented a comprehensive inventory of global marine nonindigenous amphipods and their distributions worldwide, allowing for horizon-scanning initiatives, predictive species distribution modelling, as well as NIS monitoring. The authors also highlighted a number of knowledge gaps, notably the challenges in assessing the invasion status of species with certainty owing to taxonomic problems and dubious species records (Marchini and Cardeccia 2017).

A study modelling the connectivity among metapopulations of lionfish (*P. volitans/miles*) in the Gulf of Mexico identified the Campeche Bank as an important source of lionfish recruits to the north-eastern Gulf of Mexico (Johnston and Bernard 2017). Both Miller (2016) and Johnston and Akins (2016) developed sophisticated models to forecast the spread of newly reported NIS, the ascidian *Didemnum vexillum* in Southeastern Alaska and the damselfish *Neopomacentrus cyanomos* in the Gulf of Mexico, respectively, based on physiological tolerances and/or life history traits of species. A study comparing per capita algal resource use at different temperatures among three mussel species, the recent invader *Semimytilus algosus*, the established invader Mediterranean mussel *Mytilus galloprovincialis*, and the native *Aulacomya atra*, predicted that *S. algosus* will become established along the south coast of South Africa, though *M. galloprovincialis* will maintain dominance along the coast (Alexander et al. 2015). Podbielski et al. (2016) proposed that the sea anemone *Diadumene lineata*, recently discovered in Kiel Fjord in the Western Baltic Sea, could invade the Kattegat and Skagerrak regions, but not the Baltic Proper based on the critical salinity obtained from physiological assays. Finally, a study examining the physiology, life cycle constraints, and habitat availability of the European cuttlefish *Sepia officinalis* suggested that the species has the potential to expand its range to North America via the North Atlantic under climate change (Xavier et al. 2016).

## Impacts of nonindigenous species

The dramatic effects of invasive species on recipient ecosystems are well recognized (Simberloff et al. 2013). This collection compiles information on the impact of over 40 marine NIS, including five well-known invaders, the killer alga *Caulerpa taxifolia* (Cvitkovic et al. 2017), the alga *U. pinnatifida* (South and Thomsen 2016), the European green crab *C. maenas* (Gehrels et al. 2016; Lutz-Collins et al. 2016; Quinn and Boudreau 2016), the Mediterranean mussel *Mytilus galloprovincialis* (Alexander et al. 2015), and the Asian date mussel *Arcuatula senhousia* (Como et al. 2016). The first four listed species are considered the world’s worst invasive NIS (ISSG 2016), while the latter is listed as one of the 100 worst invasive NIS in Europe (DAISIE 2016).

For example, two nonindigenous macroalgae *Schyzimenia dubyi* and *Ahnfeltiopsis* sp. recently detected on the Argentinean coast around Mar del Plata modified the benthic habitat and altered the structure and composition of benthic biota (Palomo et al. 2016). The European green crab (*C. maenas*) significantly altered the community structure of local invertebrates in muddy habitats of Prince Edward Island, Canada by creating feeding pits in sediments (Lutz-Collins et al. 2016). Comparisons of meiofauna assemblages associated with bare sediments, the killer alga (*C. taxifolia*), and the native seagrass *Posidonia oceanica* in the eastern Adriatic coast suggested that the invader altered the structure of meiofauna assemblages and caused a decline in meiofauna density (Cvitkovic et al. 2017). A laboratory experiment demonstrated that the nonindigenous Asian date mussel (*A. senhousia*) could disrupt benthic-pelagic coupling by reducing the ^13^C-uptake by the native clam *Ruditapes decussatus* and thus the availability of phytoplankton-derived C for deposit feeders (Como et al. 2016). Goren et al. (2016) reported that the food web structure of mixed native-nonindigenous fish communities in the Mediterranean Sea off the coast of Israel had undergone dramatic modifications as a result of increased dominance of nonindigenous fishes at the high trophic levels. A study quantifying losses of ecosystem services caused by the lionfish (*P. volitans/miles*) in Bahamian reefs found losses of 26.67 and 21.67 discounted service unit years (DSUY) per km^2^ owing to reductions in recruitment and biomass of lionfish prey fishes (Johnston et al. 2015).

Negative impacts of NIS; however, may be mitigated by effective prevention and management efforts (Simberloff 2008). Fiori et al. (2016) developed a spatially explicit risk assessment to evaluate the potential effects of different strategies, including integral sanitation of the coastal zone, treatment of domestic sewage, and manual removal of oyster beds at specific locations, for managing the impacts of the Pacific oyster (*Crassostrea gigas*) in the Bahía Blanca estuary, Argentina. The potential effectiveness of individual approach is expected to vary depending on the location, though all approaches would offer risk reduction to some degree (Fiori et al. 2016).

## Conclusions

Biological invasion is a major driver of global environmental change (Simberloff et al. 2013). While considerable research has been conducted to understand and predict invasions, the amount of effort devoted to these studies is not consistent across ecosystems, with the majority of the studies undertaken in terrestrial systems rather than aquatic ones (Jeschke et al. 2012; Lowry et al. 2012). This Topical Collection on Invasive Species addresses this knowledge and data gap by compiling some of the most recent research in marine invasion ecology. Articles in the collection considered a wide range of topics, including propagule pressure associated with transport vectors, species characteristics, attributes of recipient ecosystems, invasion genetics, biotic interactions, testing of invasion hypotheses, invasion dynamics and spread, and impacts of nonindigenous species. However, this collection is not by any means inclusive. For instance, the collection examined only two of the many important transport vectors of marine NIS (shipping and aquaculture). Other species traits in addition to physiological tolerance need to be investigated in future studies. Only three invasion hypotheses (ERH, NWH, and EICA) were empirically tested in studies of this collection. The inconsistent results obtained by these studies highlight the complexity and the context-dependent nature of biological invasion. Therefore, much research is still needed to advance the field.
